# On the Disease of the Hip Joint: With Plain and Coloured Plates

**Published:** 1837-01-01

**Authors:** 


On the Disease of the Hip Joint: with plain and coloured Plates. By *William Coulson*, Consulting Surgeon to the London Lying-in-Hospital; late Surgeon to the General Dispensary, &c. &c. 4to. pp. 111. 5 plates.

Mr. Coulson is known to the profession as the editor of the English translation of Blumenbach's Manual of Comparative Anatomy—to the public as the author of a very sensible work on deformities of the chest—to his friends as an industrious and intelligent surgeon. Formerly engaged in the honourable occupation of teaching human anatomy, necessarily acquainted with the interesting and instructive mass of facts which comparative anatomy presents, he brings to the investigation of disease a mind educated in the proper manner.

It might almost seem hopeless to attempt discovery in the pathology or in the treatment of diseases of the joints—diseases which have been studied by many able men, with the assistance of those lights which modern pathology and morbid anatomy afford. Yet omissions are made by the most accurate, fallacies creep into the observations of the most cautious and the most acute, and perhaps there is no disease within the pale of medicine or of surgery, on which additional inestigations will be expended quite in vain.

The affections of the hip-joint are confessedly obscure, and there has been some difference of opinion with respect to them. What may be called the "haute pathologie," of all the joints is yet unsettled, and recent attempts, it is well known, have been made, to disturb what were considered unassailable opinions. M. Coulson, then, need offer no apology for inquiring where inquiry would seem to have become requisite, nor for publishing the results of investigations, provided they have been pursued with care and reported with accuracy.

The work contains seven chapters, severally headed:—The anatomy and physiology of the hip-joint. The cause of the disease of the hip-joint. The pathology of the hip-joint. The morbid anatomy of the hip-joint in this disease. The symptoms of the disease of the hip-joint. The diseases with which that of the hip-joint may be confounded. The treatment of disease of the hip-joint.

It is unnecessary for us to do more than lay before our readers those remarks of our author which present us with new facts, or which view old ones in a new light; in short, it is only necessary to allude to what wears in some degree or other the garb of novelty. We may therefore entirely dismiss the first chapter, which discusses the anatomy and physiology of the hip-joint. The second, exposes the views of Mr. Coulson on the cause of the complaint, and as his views are in some measure peculiar, we must pause in order to exhibit them.

According to our author, disease of the hip-joint is essentially an affection of the secreting system. His principal reasons for this opinion may be readily summed up:—the disease is avowedly rather constitutional than local, and is frequently associated with scrofula—it is almost universally attended with affection of the liver—it may give rise to dropsy—and, finally, the most successful treatment operates by promoting absorption, as iodine. Children are certainly more subject to disease of the hip-joint than adults, and, perhaps, females are more liable to it than males. Now Mr. Coulson argues, that in both, the loco-motive system being weak, and the secreting powerful, the excess or derangement of the former overpowers the latter, and the hip-joint becomes a sacrifice. The view is ingenious, but it must be owned to be rather hypothetical. We need not follow it out in detail, nor apply it, as our author has done to the explanation of some well-known circumstances connected with the causes of the malady.

The pathology of the hip-joint is treated of in the third chapter, and the diligence of Mr. Coulson is displayed in his view of the opinions of his predecessors. Mr. Coulson is of opinion that the disease is essentially seated in the synovial membrane.

"The true local cause of the disease (conforming perfectly to the doctrine I have ventured to adduce, namely, that the disease is really a constitutional one,—that it is a disease of secretion,—and that it originates in the part of the joint most vascular and most liable to disease) is pointed out by Camper and Van Swieten, who attribute its origin to an unusual collection of synovia.

Petit is also of opinion that, in addition to relaxation of the ligaments of the joint, paralysis of the muscles and swelling of the head of the bone, it may arise from a collection of synovia.

That the synovial membrane of the hip-joint, and not the cartilage, is often primarily engaged in this disease, we may infer from one of the first symptoms which marks its commencement, a fulness of the groin, depending in all probability upon the increased secretion into the joint, similar to that which we know takes place in synovitis of the knee." 32.

In illustration of this view he relates a case. It was that of a child twenty months old, first seen by Mr. Coulson in April, 1836. There were then wasting of the right nates, and apparent elongation of that limb. The disease went on, and the child died in June. Setting aside other less important particulars, on opening the capsular ligament, some thick yellowish matter escaped, resembling, in consistence, the cortical substance of the brain. The quantity amounted to half a table spoonful. The round ligament was completely destroyed, and the synovial membrane inflamed.—There was absorption of some portion of the cartilage covering the head of the femur, and of the cartilaginous rim of the acetabulum; but on making a section of the femur, the bone was found healthy, and the cartilage no further diseased.

"I may mention another case which confirms the view I have taken of the origin of the disease.—With my friend Dr. Lambe, I had some time ago an opportunity of examining the body of a child six years old, who died of an inflammatory affection of the lungs, but who, at the time this illness commenced, was labouring under incipient disease of the right hip-joint. The synovial membrane was inflamed, particularly that part investing the round ligament; the red vessels extended along the ligament over the cartilage of the head of the femur; and I know of nothing with which I can so well compare the appearance of the cartilage as the conjunctival membrane of the eye in a state of incipient inflammation." 36.

The consideration of the pathology leads to that of the morbid anatomy of the hip-joint. We need not dwell on the ulceration of the cartilages, the affection of the bone, the thickening or ulceration of the capsule, the abscesses, the sinuses, nor on consecutive dislocation of the head of the femur. But we may mention the changes which ensue, when the curative process has begun, as, probably, they are less familiar than the rest. Mr. Coulson observes, that the ligaments are found, in some instances, thicker than usual, with a soft substance, which seems to have been first thrown out as coagulable lymph, but now forms a solid and organized mass, adhering to a greater or lesser portion of their internal surface; and in cases where the articular cartilages have been absorbed, this substance is found to pass between the surfaces, which when covered with cartilage moved on each other, and now to unite them by adhering to, and receiving vessels from, each; thus forming the connecting medium through which anchylosis takes place. If the capsular ligament be not destroyed, the carious head or neck of the bone is drawn towards the acetabulum, and dislocation usually does not take place. Anchylosis of the head of the bone in this situation takes place. At other times, the head of the femur is dislocated; and anchylosis takes place between the femur and dorsum of the ilium or a new joint may be formed here, and a certain degree of motion be allowed. When the head of the bone is dislocated, ossific deposit is thrown out in the acetabulum, which in time becomes almost obliterated.

The change which the bones of the pelvis undergo, has been studied and described by Albers and Rust, from whom our author has transcribed it. With this also our readers are probably unacquainted, and we shall quote the passage that portrays it.

"The pelvis, in those who have for a long time gone lame, is pushed upwards; and the sacrum is flat and straight: in a few cases, however, it is more curved than in the natural state. The coccyx is bent strongly forwards, and the connection of the last lumbar vertebra with the sacrum forms a right angle. The ilium of the affected side stands higher, and has, in general, a perpendicular direction, and more of a triangular form. The external surface is smooth, whilst the fossa appears more hollowed than usual. This hollowing probably depends on the action of the iliacus internus, which is greater than that of the glutæi. The horizontal portion of the pubes often seems lengthened, and lower than in the natural state, and the ischium is usually drawn outwards and forwards. The perpendicular direction of the foramen ovale is changed more to a horizontal one, and the opening assumes more of a triangular form, its base being turned to the acetabulum. In consequence of the changed situation of the bones of the pelvis, its different diameters undergo an essential deviation from the natural state. The superior apertures of the pelvis are commonly somewhat oblique: and the pelvis is broader on the affected side from before backwards.

Dissections of those persons who have walked lame for a long time in consequence of this complaint, show, that the muscles have been exercised in a different direction from the natural one. Some are relaxed, particularly the three glutæi, which are quite drawn together and shortened. In addition, the anterior part of the glutæus medius and minimus is drawn backward, and the back part is pressed, in an almost straight direction, on the trochanter. Other muscles are drawn forward with the dislocated limb, as the obturators. The gemini, the pyriformis, and quadratus, become shortened, as the femur is drawn nearer to their attachment. Other muscles, from similar causes, are drawn inwards; and this is the case with the psoas and iliacus internus. The triceps, gracilis, and pectinæus are drawn outwards. Under the tendon of the rectus femoris, which is drawn backwards, the head of the femur ascends. Sometimes several muscles are found entirely hardened by the preceding suppuration, joined together, and converted into one tendinous mass. The sciatic and crural nerves are found on the stretch or pushed to one side. Sir B. Brodie mentions a case in which he found two enlarged lymphatic glands, each of the size of a walnut, immediately below the crural arch, on the fore part of the joint; and these lay in contact with, and immediately behind, two branches of the nerves, so as to keep the latter on the stretch, like the strings passing over the bridge of a violin. The hip, therefore, of the affected side is much less prominent, or rather, is much flatter than the other, and is often intersected by sinuses discharging very fetid purulent matter." 44.

Mr. Coulson dwells, of course, on the symptoms of this disease, symptoms at times obscure, physiologically interesting, but too familiar, from the many excellent accounts which have been given of them, to permit us to speak of them at any length. Yet as our author offers a new explanation of that symptom, which has always attracted the attention, and indeed excited the curiosity of surgeons, we think we may advert to it. We allude to the pain in the knee, which is frequently, though not always, felt in the early stages of hip-disease. It is commonly supposed to depend on the inflammation of the capsule, affecting the anterior crural nerve which lies upon it. Sir C. Bell has attributed the phenomenon to implication of the obturator nerve, which sends a twig or two into the hip joint, and is finally distributed upon the inside of the thigh. Mr. Coulson hints another opinion.

" It has struck me," he remarks, " that, from the intimate connection of the long head of the rectus femoris with the outer edge of the acetabulum, and with the capsular ligament, this muscle may take on the inflammatory action, and the pain in this way be conveyed down the limb to the thigh. We find something analogous to this in diseases of the shoulder-joint: the pain in these cases extends down the front of the arm to the insertion of the biceps, and the long head of the biceps is in intimate connection with the capsular ligament of the joint, and the glenoid ligament of the scapula." 48.

It appears to be an objection to this view, that we know of no reason why inflammation at the origin of a muscle, should cause pain at its insertion. We are not aware of any facts which establish such a general law. But irritation or inflammation of a nerve at its source, or in its course, does, it is well known, occasion pain to be referred to its cutaneous terminations, and occasionally, though rarely, to its origin. The case of inflammation of the testicle offers an illustration of both these facts; it is not uncommon for the patient to feel pain on the surface of the hip, *and* in the loins. The pain in the hip is seated in the cutaneous ramifications of the lumbar nerves in connexion with the spermatic: and the pain in the loins is at the origin of the spermatic nerves themselves. In the cases we have seen of disease of the shoulder-joint, the pain has either been referred to the inner side of the elbow, in the course of the internal cutaneous nerves, or has been felt apparently in the bone itself.

Passing over the *apparent* lengthening of the limb affected, which is well known to occur early in the disease, and merely glancing at the most usual cause of the *real* shortening which occurs in the later stages, we allude to consecutive dislocation of the head of the femur on the dorsum of the ilium, we may observe that this shortening *may* be independent of any dislocation whatever.

"The real shortening often occurs without dislocation of the femur having taken place. A case of this kind occurred to me, some time ago, in the General Dispensary. A girl of the name of Dexter, who had been for a long time under my care, died with disease of the hip in the most advanced stage; there was shortening of the limb, prominence of the nates, and abscesses in the neighbourhood of the joint. All these symptoms led me to conclude, that there was dislocation of the head of the femur; but, on carefully examining the joint after death, I found the capsular ligament entire, and no dislocation. The head of the bone, and a great part of the neck, had been destroyed by caries, and in this way the shortening was produced without dislocation." 58.

We have seen two instances of shortening of the limb, occasioned in another manner. The head of the femur was partly destroyed by ulceration, and the bottom of the acetabulum was removed by the same process. What remained of the head and neck of the bone had passed through the aperture at the bottom of that cavity, and had consequently entered the pelvis.

Dislocation of the head of the bone has occurred into the ischiatic notch. Such a case is mentioned by Mr. Earle. In a preparation in the Museum of the College of Surgeons, the head of the femur is dislocated into the foramen ovale. Boyer relates a case of this sort. Sir Benjamin Brodie alludes to another. The following is an outline of a case which occurred to Mr. Hides, of Emsworth.

*Case.* "Master S., æt. nine years, in 1826 laboured under a severe affection of the hip, accompanied with great constitutional irritation. Matter formed in front of the joint, for the evacuation of which an opening was made. The wound continued to discharge for a long time, and pieces of bone occasionally came away. In the middle of August 1829, the child had a fresh attack of the disease of the joint. On the 8th of October he was examined by Mr. Hicks, who found the limb much elongated, the knee and foot turned outwards, and the head of the femur near, or in, the foramen ovale. By counter-irritants, rest, and attention to the general health, the complaint in the hip was arrested, and the child restored to perfect health : the deformity, of course, remains. The nates, which were flat, or even flabby, are now become rounded or prominent, and swollen, and the toes are turned inwards." 61.

The sixth chapter is occupied with a consideration of the cases of other diseases which may be confounded with disease of the hip. They are, congenital displacement of the hip-joint—congenital mal-position of the acetabula, usually caused by rickets—unnatural shortness of the psoas and the internal iliac muscles?—unequal length of the two lower limbs—necrosis of the upper end of the femur—deep-seated abscess in the groin—psoas abscess—malignant tumor situated over the trochanter major—inflammation and effusion in the bursa placed between the tendon of the iliacus and psoas, and the capsule of the hip-joint—disease of the sacro-iliac synchondrosis—caries of the spine—sciatica—an affection of the hip occurring about the period of dentition—nervous affection of the hip in hysterical females.

In most of these cases an accurate diagnosis can only result from an accurate knowledge of the various diseases which simulate that of the hip. But we will advert to one or two of the former.

1. Mr. Coulson properly observes that the diagnostic marks between psoas abscess and the disease of the hip-joint have been thus arranged:—First, in psoas abscess, the patient complains of violent or dull pain in the region of the loins, which is very much increased in the upright posture of the body, and every motion of the limb, particularly on extending it: in the diseased hip, there is no fixed pain in the loins; it is felt more in the neighbourhood of the hip, and especially in the knee. Secondly, in psoas abscess, during the whole course of the complaint, there is no deviation to be perceived in the natural situation of the trochanter, and no difference in the length of both limbs; in diseased hip, on the contrary, this is always the case. Thirdly, in the affection of the psoas muscle, the patient cannot turn the foot of the affected side outwards, without increasing the pain : in diseased hip, on the contrary, the foot is generally turned outwards. Fourthly, on taking a deep inspiration, on coughing, crying, and in the erect posture of the body, the fluctuating swelling either on the nates or on the front of the thigh increases, and the exit of the matter, if the abscess be burst or opened, will be facilitated : in abscess of the hip-joint from disease, neither is the case.

We would observe that these diagnostic symptoms are not always sufficient to enable us to discriminate the two diseases. In cases of disease of the lumbar vertebræ, or of what is usually the same thing, psoas abscess, prior to the appearance of suppuration externally, the patient occasionally complains of lameness, and of pain in one hip. Nay, he will do what we are assured he never does—he will display apparent elongation of the limb which is the seat of suffering. Probably the pain in the hip is occasioned by inflammation at the root of the anterior crural nerve, where it lies in the psoas muscle—the lameness has perhaps a similar cause—and the apparent elongation of the limb results from the patient *favouring* it, as he does when the hip-joint itself is affected.

We lately had a gentleman under our care, in whom these symptoms existed. Yet when we forcibly pressed the head of the femur into the socket no pain was occasioned, and we suspected that the joint was not diseased. We made the patient flex the thigh by his own effort—he felt pain in the groin. We extended the thigh on the pelvis by force—he had pain in the groin and in the hip. We elongated the limb—he had pain. This led us to think, for physiological reasons that cannot but be obvious, that the mischief was in the lumbar vertebræ, or in the psoas muscle. We therefore made a careful examination of the back, and discovered slight tenderness on pressure in the lumbar region. We expressed our opinion that the disease was there, and so it finally turned out—for, many months afterwards, an abscess presented at the side of the upper lumbar vertebræ.

2. In an affection, says our author, rarely occurring, namely, inflammation and suppuration in the bursa, beneath the conjoined tendons of the psoas and iliacus, it is observed, that besides the absence of acute pain on jarring the articular surfaces, by compression at the sole of the foot, if we cause the patient to lie on his belly, we do not see that flattening of the buttock, and prominence of the trochanter, so characteristic of hip-joint disease.

We lately had a case of this kind under our care. A very excellent surgeon residing in Arundel, Mr. Fletcher, having suffered much from rheumatism, and being afflicted with much pain in the right hip and with lameness, was pronounced by some of his medical friends, at Chichester, to labour under disease of the joint. He came to town, and we examined the hip very carefully. The nates were wasted—there was apparent shortening of the limb—and there was pain on walking and on moving the articulation. A more particular investigation shewed that there was no pain on pressing the articulating surfaces together, whilst there was pain on pulling at the limb, on twisting the capsule, or on forcibly extending the limb on the pelvis. In front of the joint, in the situation of the tendon of the psoas and iliacus, there was a perceptible thickening, and some tenderness on pressure. We concluded that the disease was chronic rheumatic inflammation of the bursa under the tendon in question, and probably of the fibrous tissues in the neighbourhood. We had the pleasure of meeting Sir Benjamin Brodie on the case, and he took a similar view of it. The progress of the complaint has amply proved that the joint was sound.

3. Mr. Coulson observes that a mother may bring to a surgeon a child with supposed disease of the hip: on inquiry, he learns that the child could walk at the usual period, but that, when eighteen or twenty months old (or even at an earlier age), he was unable to stand, and that the child was at this time cutting the teeth. On examination of the limb, we find it wasted, the nates of the affected side flat, and the limb, if there be any difference, a little longer than the sound one. When the child attempts to walk, it cannot raise the limb from the ground, but draws it along; and when it stands, the weight of the whole body is rested on the sound limb, whilst the limb of the affected side is half bent.

The diagnostic mark of this disease is the absence of any pain in the joint. If we place the child on the table, and press in the neighbourhood of the joint, or rotate the head of the femur, no pain is produced: whereas, in disease of the hip, the pain would be great. Some years commonly elapse before the child recovers the use of the limb, but after the period of dentition, the general health suffers little from the disorder. It is a pity that we have not the information which dissection might give us on its nature.

The last chapter is naturally dedicated to the important subject of treatment—that subject which as certainly forms the conclusion of a medical treatise, as a marriage does of a novel. Perhaps they are often equally happy.

Mr. Coulson first adverts to the treatment of the more simple and acute cases of inflammation of the hip-joint, and then to the management of that usually constitutional form, which has been emphatically though loosely termed the "morbus coxarius." On the former head we need say nothing, on the latter, we may introduce a remark or two.

1. In this, as in many other forms of constitutional disease, we find that pure air, and especially that of the sea-side, will often do more than medicine or the surgeon. The stage of this disease adapted for the removal of the patient to the country, is not that in which there is acute inflammatory action, or in which matter is forming. It is when the symptoms are moderate, and disposed to be chronic. Warm salt water bathing usually agrees. The season for the sea-side is, of course, the Summer—from the beginning of May to the end of October, provided the cold weather does not set in earlier. Dr. Brown told Mr. C. that, when the cold sets in, the strumous sores assume, in all cases, a very unhealthy, and in some cases a sloughy, appearance. The plan adopted at the infirmary is as follows:—The patient commences with the warm salt-water bath, about three times a week, at a temperature of 96°, and is directed to remain in it from fifteen to twenty minutes each time. Afterwards, the tepid bath is used; and then, dependent on the state of the weather and the health of the patient, the cold bath is employed, one dip only in the sea being allowed each time. The time selected for bathing is in the morning. The cold or warm douche bath is often used in this stage of the complaint, and with very good effect.

2. It has been questioned whether rest is indispensable in all cases. Most assuredly it is not. When exercise occasions no pain, and induces no inflammatory action, it may safely be permitted. But the case in which it may be so, and the amount that may be taken, must be determined by the surgeon, on whose caution and judgment a heavy responsibility will lie. On the whole, there can be little doubt that passive exercise is preferable to active, and that if there is to be an error, one on the side of rest is the safest.

Rest of the part may be in some degree obtained, even when general rest is not enjoined. This has been effected by pasteboard splints and bandages, which are not so well adapted for the hip as for other joints—by the method recommended by Mr. Scott—or by a sort of case of lint, soaked in a solution of gum which hardens. Mr. Scott's mode of "doing up" a joint is so useful on many other occasions, that we extract an account of it for the benefit of those who may not be familiar with it. Many of our readers, no doubt, are so—some, however, are not, and the quotation will be useful to the latter.

"The surface of the joint," says Mr. S., "is to be carefully cleansed by a sponge, soft brown soap and warm water, and then thoroughly dried. Next, this surface is to be rubbed by a sponge soaked in camphorated spirit of wine, and this is continued a minute or two, until it begins to feel warm, smarts somewhat, and looks red. It is now covered with a soft cerate, made with equal parts of the ceratum saponis, and the unguentum hydargyri fortius cum camphorâ. This is thickly spread on large square pieces of lint, and applied entirely round the joint. Over this, to the same extent, the limb is to be uniformly supported by strips of calico, spread with the emplastrum plumbi of the London Pharmacopœia. These strips are about one inch and a half broad, and vary in length: some are fifteen inches; others, a foot; others, half these two lengths; and the shorter or longer are selected, according to the size of the part round which they are to be applied. This is the only difficult part of the process. This adhesive bandage ought to be so applied as to preclude the motion of the joint, and prevent the feeble coats of the blood-vessels from being distended by the gravitation of their contents in the erect posture, and thereby promote their contraction. Over this adhesive bandage, thus applied, comes an additional covering of emplastrum saponis, spread on thick leather, and cut into four broad pieces, one for the front, the other for the back, the two others for the sides of the joint. Lastly, the whole is secured by means of a calico bandage, which is put on very gently, and rather for the purpose of securing the plaster, and giving greater thickness and security to the whole, than for the purpose of compressing the joint. This is an important point, as otherwise an application which almost invariably affords security and ease may occasion pain, with all its attendant mischief." 84.

Of course, such a plan is not adapted for the case of active inflammation, of active effusion, of open abscesses, nor of abscesses actually forming. But it is excellently suited to very chronic cases, where warmth, support, and rest are calculated to be serviceable.

3. In a constitutional disease, constitutional remedies are, of course, indicated. On the general principles which should guide us in our treatment of scrofula, it would be idle for us to dwell. But latterly the iodine and the hydriodate of potass have been much employed in the management of scrofula. We confess that we think they have both been overrated, and that they are inferior to judicious combinations of steel, the liquor potassæ, and sarsaparilla. It would appear from the following passage, that the experience of the officers of the Sea-bathing Infirmary, has led to similar conclusions.

"Iodine has been very extensively used, during the last three years, at the infirmary, and the formulæ employed are those recommended by Lugol; varying according to circumstances. Latterly Dr. Canham, one of the physicians to the infirmary, informs me that the use of this remedy has been more confined to its external application than at an earlier period of its use; as in many cases its absorption has been rapid, and constitutional effects have been soon produced, in which case the internal administration of it has been suspended. Patients are met with, occasionally, who will not bear the external application of iodine in any form, even for a short time, without its producing constitutional effects; the symptoms of which are generally an increased velocity of pulse, very foul tongue, head-ach, pains in the back and limbs, thirst, loss of appetite, and the general symptoms of pyrexia." 102.

We must now part from our author. We have not attempted to go methodically through the history, symptoms, and treatment of diseased hip-joint. We have so fully and so lately considered the various affections of the articulations, that it is impossible for us at present to break up that ground again. Mr. Coulson has spared no pains in making himself acquainted both with the literature and the actual characters of the important complaint we now take leave of.

